# Fostering the Catalyst Role of Government in Advancing Healthy Food Environments

**DOI:** 10.15171/ijhpm.2018.10

**Published:** 2018-02-12

**Authors:** Raphael Lencucha, Laurette Dubé, Chantal Blouin, Anselm Hennis, Mauricio Pardon, Nick Drager

**Affiliations:** ^1^School of Physical & Occupational Therapy, McGill University, Montréal, QC, Canada.; ^2^McGill Centre for the Convergence for Health and Economics, McGill University, Montréal, QC, Canada.; ^3^Institut national de sante publique, Québec, QC, Canada.; ^4^Department of Noncommunicable Diseases and Mental Health, Pan American Health Organization, Washington, DC, USA.; ^5^Pan American Health Organisation, Washington, DC, USA.; ^6^McGill University, Montréal, QC, Canada.

**Keywords:** Non-communicable Disease, Food Industry, Government, Multi-stakeholder Partnership, Governance

## Abstract

Effective approaches to non-communicable disease (NCD) prevention require intersectoral action targeting health and engaging government, industry, and society. There is an ongoing vigorous exploration of the most effective and appropriate role of government in intersectoral partnerships. This debate is particularly pronounced with regards to the role of government in controlling unhealthy foods and promoting healthy food environments. Given that food environments are a key determinant of health, and the commercial sector is a key player in shaping such environments (eg, restaurants, grocery stores), the relationship between government and the commercial sector is of primary relevance. The principal controversy at the heart of this relationship pertains to the potential influence of commercial enterprises on public institutions. We propose that a clear distinction between the regulatory and catalyst roles of government is necessary when considering the nature of the relationship between government and the commercial food sector. We introduce a typology of three catalyst roles for government to foster healthy food environments with the commercial sector and suggest that a richer understanding of the contrasting roles of government is needed when considering approaches NCD prevention via healthy food environments.

## Introduction

A comprehensive approach to non-communicable disease (NCD) prevention requires actors to align their actions. NCD prevention largely hinges on healthy consumer environments, with the key risk factors for the recent rise in the incidence of NCDs being commercial products such as tobacco, alcohol and unhealthy foods.^1,2^ Tobacco control interventions targeting the consumer environment such as smoke free spaces, advertising bans, and legal age limits, have restricted access to tobacco products and changed social norms with dramatic reductions in population-level consumption.^3,4^ This same logic is often taken up in efforts to shape consumer food environments.^5^

Government plays a critical role in shaping food environments, through interventions that promote the economic performance and competitiveness of the agri-food sector, public health interventions that control the nature and types of foods available to consumers and interventions that seek to influence consumer food choice. Governments have been most comfortable implementing information-based public health interventions. For example, in addition to extensive use of social marketing and other education programs, the use of nutrition labelling on packaged, processed and pre-prepared foods has become common practice for governments around the world. Other interventions have involved attempts to control the content of food products, such as policies banning transfat or restricting the levels of sodium in foods. However, these interventions have faced significant opposition, including most prominently resistance from the food industry. This opposition often results in the implementation of non-enforceable and voluntary standards. Recent proposals encourage the employment of fiscal policies that impose price measures on products deemed unhealthy, such as sugary beverages.^6,7^ Not surprisingly, these types of measures continue to be met with massive opposition from the food industry.

Despite the often tenuous relationship between government and the commercial sector, there is wide recognition that to foster healthy food environments there must be a deep engagement amongst all relevant actors. Navigating the tenuous relationship between government and the commercial sector is at the core of the Whole-of-Society^8,9^ approaches underlying the United Nations recommendations to curb the progression of NCDs and associated risk factors,^2,10^ and more recently the Sustainable Development Goals.^11^ This push to foster relationships between government and the commercial sector is largely captured in the discourse on multi-stakeholder partnerships (MSPs), seen by some as a way for partners to leverage unique strengths to foster more effective and wide-reaching interventions.

Despite the recognition that MSPs are an important strategy to address NCD prevention through intersectoral action, they remain highly controversial. The principal controversy remains whether governments can work with commercial interests in a way that does not sacrifice public interests to private ones, and whether private interests can serve the public good. In other words, to what extent are MSPs vulnerable to shifts away from the public health agenda to special interests and do these interests run counter to the collective good? Controversies are often centered on whether governments can develop sound policies with the public interest in mind, particularly as regulators of the market, while in partnership with the private sector.^2,12,13^ This type of controversy is salient in a context where large multinational companies actively resist being regulated.^2,14,15^ Given that commercial interests do not always align with the public interest and in light of evidence that many of the “vectors” of NCDs are products actively promoted by industry, the regulatory role of governments must be protected from the detrimental influence of such interests.^16,17^ In fact, it is argued with good reason that government should never partner with companies that produce products known to cause harm to human health, with the most obvious being tobacco companies.^18^ From our perspective, the crux of government is how to regulate health harming products while simultaneously promoting consumer environments that make healthy food choices easy and accessible.

In addition to the regulatory role of governments, it is well documented that governments utilize other tools in their relationship with the commercial sector to foster healthy food environments. Governments can and do play an important role in shaping the supply and demand for nutritious food. There is an ongoing need to scale-up efforts to promote healthy food environments when almost all food consumed in high-income countries, and increasingly in low- and middle-income countries, is procured from commercial sources.^19,20^ In other words, governments must not only control the proliferation and severity of unhealthy foods, they must also promote companies that are making positive strides to build healthier food environments.

In this article, we focus on the different tools that government can use to serve as catalysts for healthy food environments. A healthy food environment is defined in this paper as one in which healthy foods (eg, fruit and vegetables) are accessible, attractive and affordable. The objective of this paper is to distinguish between the regulatory and the catalyst roles of government. We then discuss how these roles can be managed to ensure optimal outcomes in the context of MSPs for NCDs prevention by fostering healthy food environments.

## Government as Regulator

There are numerous generic classifications of the tools used by governments. Vedung offers a simple classification of government instruments: regulation (“stick”), economic means, such as incentives or inducements (“carrot”) and information (“sermon”).^21^ Hood’s^22^ typology, uses a similar framework but also includes “organization,” which he describes as the “capacity (of government) for direct action, for instance through armies, police or bureaucracy” (p. 129). We present these two classification schemes to demonstrate that there has been, and continues to be, a large body of work articulating the many ways that government engages with market and society beyond the traditional role of regulator.^23^ The role and tools of government continue to be thrown into flux, particularly with the dominance of market oriented theories of society. As noted above, the public health literature has focused to a great degree on the regulatory (ie, “stick”) role of government and the need to protect the autonomy of this function from private interests. It is critically important to enrich the discourse on the role of governments in shaping healthy food environments by articulating and analyzing the other roles played by government.

Regulation of products and services remains a crucial role of government. Law is an important tool to create healthy consumer environments.^24-26^ Governments have achieved successes towards reducing the incidence of NCDs by regulating products that are particularly harmful to human health such as tobacco and alcohol. This regulatory role is also indispensable when it comes to ensuring the safety of medicines and other products used to treat disease and establish laws pertaining to safe practices such as the use of protective equipment such as bicycle helmets.

The regulatory role has been particularly indispensable given the history of deceit by certain product manufacturers. The case of tobacco is the most dramatic example of a product which was widely and aggressively promoted in the absence of government regulation to the detriment of human health. Parallels have been drawn between this history of unregulated tobacco promotion and the contemporary context of the largely unregulated promotion of unhealthy food products especially to children, and the need for statutory regulation by governments.^27^

The dominant discourse about the role of government in addressing food-related NCDs centers on how the state can best regulate the food information environment. An intense debate continues about whether food information and food content should be controlled through legislative means or through standards directed at industry self-regulation.^28-30^

When focusing on the regulatory role of public health authorities, clear and strong mechanisms to protect the autonomy, accountability and transparency of state actors involved in law-making are needed. Most governments do indeed consult with stakeholders in the law-making and regulatory process. However, part of the legitimacy of state actors is based on the premise that no one group or organization has privileged access or undue influence over government decision-making, for example the adoption of food taxes.^31^

Regulatory capture, the positioning of private special interests in place of public interest in public policy, is a pressing and perennial challenge for public institutions.^32^ This phenomenon explains why the concerns over conflict of interests have remained salient in the media and in research about the role of government in addressing NCD prevention.^13,15^

Concerns regarding the independence and legitimacy of government regulation are so prominent that the World Health Organization (WHO) developed a Framework of Engagement with Non-State Actors, which clearly outlines rules about engagement with non-State actors and identifies measures to prevent undue influence.^33^ It is most clearly stated in a statement by the former Director General of the WHO^34^: “WHO may engage with the private sector on occasion, but according to WHO policy, funds may not be sought or accepted from enterprises that have a direct commercial interest in the outcome of the project toward which they would be contributing. For this reason, the Organization does not accept funding from the food and beverage manufacturers for work on NCD prevention and control.”

As mentioned earlier, when consumers acquire most of their food through commercial channels, even in emerging economies,^35^ there can be major benefits for government to serve not just as regulators or norm-setters, but also as catalysts for change in the consumer food environment. The next section discusses the key features of this catalyst role. The regulatory role only harnesses part of what government can contribute to healthy food environments.^36,37^

## Government as Catalyst: Typology

Governance is ultimately concerned with creating the conditions for ordered rule and collective action. The outputs of governance are not therefore different from those of government. It is rather a matter of process.^38^

To begin examining how governments can facilitate healthy food environments, we introduce three different catalyst roles government can play in MSPs: (1) gathering, interpreting and sharing information; (2) facilitating a hybrid form of coordination, and; (3) providing or mobilising financial resources. Examples are drawn from a strategic analysis of a purposive sample of MSPs commissioned in 2013 by Pan-American Health Organization as part of policy dialogue programs.

### Gathering, Interpreting and Sharing Information

We borrow Hood’s^39^ idea of “nodality” to point to the “capacity of government to operate as a node in information networks.” Governments are often well situated to facilitate information sharing and generation. Governments can capitalize on the rapid rise in information and communication technologies (ICTs) to facilitate healthy food production and access. Many have worked to conceptualize the role of government in the era of social media and “big data.” Although this literature is too vast to engage with in any meaningful way in this article, we draw on one thread from this literature pertaining to the new opportunities for government to utilize information platforms to facilitate deeper webs of action among different stakeholders. Governments have the potential to serve as a credible source of information that links consumer and commercial practices in order to shape these practices. This information role does not exist in a vacuum but should be tied to a specific objective, project or program.^40^ For example, governments can play an important role in gathering and sharing information on the factors that shape fruit and vegetable consumption in a neighborhood, which can feed into actions to foster increased density of fruit and vegetable vendors. As Bodor and colleagues^41^ find, proximity to small food stores and the space dedicated to fresh vegetables in these retail outlets both served as predictors of consumption. This type of information in a specific government jurisdiction can be used to coordinate and shape the retail orientation of vendors.

Another example is large scale information sharing in the European Union (EU) Platform for Action on Diet, Physical Activity and Health. Operating under the leadership of the EU, platform members – such as groups, associations and alliances from social, sports, media, agriculture, food and public health sectors – have agreed to share information and action plans with each other to encourage national, regional, and local initiatives across Europe. Perhaps more importantly, government impacts the nutritional quality of food supply and demand through the development and dissemination of information, norms, or coding systems that target both education to empower consumers to make healthy choices, and actors from the agriculture, food and health sectors that altogether define the nutritional quality of supply and demand in the food environment.^42^

### Hybrid Forms of Coordination

Governments can play a catalyst role in facilitating new institutional forms of coordination not only between public and private actors, but also by building synergy with social innovators in non-government organisations, especially when they act as brokerage organizations in the creation of MSPs for prevention of NCDs that are comprehensive, integrated and community-based. Some, like Agita Sao Paulo, promote physical activity through public service transportations and others, have spread within the country boundary. Others like EPODE (‘**E**nsemble **P**révenons l’**O**bésité **D**es **E**nfants’), engaged municipal actors from social and commercial sectors in promoting healthy lifestyles and environment, including but not limited to food, have spread in a franchise-like mode around the world.^43^ These community-based MSPs typically serve as brokers in engaging local leaders from different sectors to address diet and physical activity in individuals and families, with supportive programs and socio-environmental transformation in agriculture, education, sports, transportation, media, health, industry, commerce and service. In its simplest sense, brokers are actors that bridge gaps in the social structures^44-46^ and facilitate the flow of goods, information and opportunities across those gaps.^47^ In this way, brokerage organizations can create new connections between previously unconnected actors.

At the global level, hybrid forms of coordination have taken the form of partnerships such as AgResults, which is a partnership between the governments of Canada, Australia, the United States, the Bill and Melinda Gates Foundation, World Bank, and the commercial sector. With a strong focus on building capacity and leadership at country level and aiming for collaboration around a common goal, partners bring their different skills and experience to participate in strategy, policy-setting, advocacy, fundraising, product development and procurement, country support and delivery. Part of the strategy is to use “pull” mechanisms to foster result-based financial incentives rewarding successful commercial and social innovations that address health and other social problems in a way that is financially sustainable and support economic development. In these, income can be accrued to social entrepreneurs and private sector partners from philanthropic and public funding upon accomplishment of specific deliverables that contribute to the health objectives of the partnership.

### Financial Resources Provision and Mobilizations

One of the ways that government agencies can serve as a catalyst is through the provision or mobilization of funds for MSPs that they have initiated (eg, Food Trust in Philadelphia, USA), that have emerged from civil society organization leadership (eg, Quebec en Forme in Quebec, Canada), or from grassroots entrepreneurship (eg, Wholesome Wave across the United States). For instance, the Philadelphia Department of Public Health (with other states and federal departments) are lead policy partners in the Food Trust, a non-profit MSP devoted to access and affordability of nutritious food and information to make healthy choice to prevent obesity and NCDs. The Food Trust works with community organizations, schools, grocers, farmers and policy makers to invest in the corner stores in underserved neighborhoods. Their most recent innovation consists of a public-private partnership creating a US$30 million fund to leverage private investment to offer grants and loans to supermarkets and grocery stores located in underserved areas. This inspired the country-level Healthy Food Financing Initiative launched by the Obama Administration in 2010, which provided $400 million towards bringing grocery stores and healthy food retailers to deprived communities. Results of this initiative are at the same time encouraging and sobering. While Philadelphia has been one of the first cities announced by the Centers for Disease Control and Prevention (CDC) as having stopped the progression of the rise in obesity rates among children, results of a case control study conducted in Philadelphia found that for those communities that benefited from the supermarket and grocery store funding program, there was no measurable effect on fruit and vegetable consumption (the target of nutritional dietary improvement) nor on body mass index (BMI).^48^ Similar results were found for a supermarket introduced in Pittsburgh, Pennsylvania.^49^

The second type of MSP is Québec En Forme, created as the deployment arm of a unique partnership launched in 2006 and spearheaded by the Quebec Public Health Directorate, bringing a lead Canadian philanthropy (The Lucie and André Chagnon Foundation) with governmental signatories. Equal investment of both parties secured a yearly CAN $48 million funding for a decade in a WoS action plan to promote healthy lifestyle and environment, with 75% of the investment to be deployed through local communities in solutions suited to their respective needs and possibilities. Ten years into this initiative, the progression of childhood obesity has shown signs of plateauing and Quebec-en-forme created as an ad-hoc transitional organization that has now morphed into community-level collective impact partnership with direct engagement and investment for community and governmental actors at city- and state-levels, with still very little private sector engagement.

Wholesome Wave in the United States taps into agriculture funds for food stamps to improve, in partnership with a rich diversity of partners from local communities and food system, the access and affordability of fresh fruit and vegetables to address obesity and NCDs in underserved communities. It does so by building capacity and fostering linkages between vulnerable populations and local food systems, while weaving behavioral economics principles to design incentives for both buyers and producers. Beyond governmental food stamps, complementary funding comes from individuals as well as local, state, national and global private sector and philanthropy. Their most recent innovation attempts to facilitate disease prevention in the healthcare system with fruit and vegetables prescriptions to adolescents struggling with obesity and mothers with diabetes from underserved communities. Together with government regulation of unhealthy foods, this approach appears promising. While rigorous research of the health outcomes is yet to come, this model and the entrepreneurship of Wholesome Wave leader has inspired the Food Insecurity Nutrition Incentive Program (FINIP), established by the Agricultural Act of 2014 to provide $100 million in federal grants between 2014 and 2018 to eligible entities that implement nutrition incentive programs. These programs provide individuals enrolled in the Supplemental Nutrition Assistance Program (SNAP, or “food stamps”) with a monetary incentive when they purchase fruit and vegetables at participating retail venues.

## Conclusion

We suggest that there is a need to extend the debate on the instruments used by governments to facilitate collective action for NCD prevention. Specifically, we propose to broaden the definition of what government can do to foster collective action towards healthy food environments. This is what we call the catalyst role of government to institutionalize new paths of convergence for NCD prevention.

The catalyst role of government can take many forms and levels of involvement. State agencies can serve as members of networks or partnerships, or take a role of the structuring or management of networks.^38^ Abbott and colleagues^50^ propose that governments can serve as “orchestrators” to facilitate movement towards a “joint governance goal.” They suggest that states can contribute to collective action by providing material and ideational support. Material support “strengthens the … operational capacities” while ideational support “such as guidance, formal approval or political endorsement, enhances the … effectiveness and legitimacy vis-à-vis targets.”^51^ A WoS approach for NCD prevention demands a high level of diversity and innovation in governance.^9,51^ It is important to restate that these catalyst roles are not meant to supplant the regulatory role of government. The regulatory role remains critical in NCD prevention, and much work needs to be done to expand the ability of government to control unhealthy food products. We draw attention to the catalyst role in order to stimulate discussion on how government can foster healthy consumer food environments while continuing to control unhealthy foods.

The catalyst and the regulatory roles of government can and do co-exist. Given that most of the levers for change in the food system remain in the hands of private actors, an effective approach to create healthy food environments will require governments to foster healthy product production. Existing and new mechanisms can be put in place to ensure that engagement in the catalyst role does not impede the autonomy, accountability and transparency of government to effectively pursue their role as regulators. Future work on MSPs should therefore not only explore how state-actors can best engage in the catalyst role. It should also examine how to implement effective mechanisms to prevent partners from gaining privileged access or undue influence on public actors when they engage in their regulatory role.

## Acknowledgements

The ideas and examples presented in this paper were partly drawn from a strategic brief commissioned by the Pan American Health Organization entitled “Scaling up multistakeholder partnerships for non-communicable disease prevention and control: Threading the needle between conflict and convergence of interests” published in 2013 (research contract 120930). Complementary funding for research and manuscript editing comes from IDRC (#107400-006), SSHRC operating grant (#435-2014-1964) and FRQSC team grant (#2015-SE-179342) to Laurette Dube. Raphael Lencucha is supported by a Research Scholar Award from the Fonds de Recherche du Québec.

## Ethical issues

Not applicable.

## Competing interests

Authors declare that they have no competing interests.

## Authors’ contributions

RL, CB, and LD developed the main ideas of the paper. RL and CB wrote the first draft of the paper with input from LD. LD provided case examples used to inform the catalyst typology. AH, MP, and ND contributed to the overall conceptualization of the manuscript.

## Authors’ affiliations

^1^School of Physical & Occupational Therapy, McGill University, Montréal, QC, Canada. ^2^McGill Centre for the Convergence for Health and Economics, McGill University, Montréal, QC, Canada. ^3^Institut national de sante publique, Québec, QC, Canada. ^4^Department of Noncommunicable Diseases and Mental Health, Pan American Health Organization, Washington, DC, USA. ^5^Pan American Health Organisation, Washington, DC, USA. ^6^McGill University, Montréal, QC, Canada.

## References

[1] Flegal KM, Carroll MD, Kit BK, Ogden CL (2012). Prevalence of obesity and trends in the distribution of body mass index among US adults, 1999-2010. JAMA.

[2] Moodie R, Stuckler D, Monteiro C (2013). Profits and pandemics: prevention of harmful effects of tobacco, alcohol, and ultra-processed food and drink industries. Lancet.

[3] Chaloupka FJ, Yurekli A, Fong GT (2012). Tobacco taxes as a tobacco control strategy. Tob Control.

[4] Widome R, Samet JM, Hiatt RA (2010). Science, prudence, and politics: the case of smoke-free indoor spaces. Ann Epidemiol.

[5] Swinburn B, Kraak V, Rutter H (2015). Strengthening of accountability systems to create healthy food environments and reduce global obesity. Lancet.

[6] Backholer K, Sarink D, Beauchamp A (2016). The impact of a tax on sugar-sweetened beverages according to socio-economic position: a systematic review of the evidence. Public Health Nutr.

[7] Le Bodo Y, Paquette MC, De Wals P. Sugar-Sweetened Beverage Taxation Logics and Ethical Concerns. Taxing Soda for Public Health: A Canadian Perspective. Cham: Springer International Publishing; 2016:75-82.

[8] Dube L, Pingali P, Webb P (2012). Paths of convergence for agriculture, health, and wealth. Proc Natl Acad Sci U S A.

[9] Dube L, Addy NA, Blouin C, Drager N (2014). From policy coherence to 21st century convergence: a whole-of-society paradigm of human and economic development. Ann N Y Acad Sci.

[10] UN. Political declaration of the high-level meeting of the General Assembly on the prevention and control of non-communicable diseases. New York, NY: United Nations; 2011.

[11] Nilsson M, Griggs D, Visbeck M (2016). Policy: Map the interactions between Sustainable Development Goals. Nature.

[12] Magnusson RS, Patterson D (2014). The role of law and governance reform in the global response to non-communicable diseases. Global Health.

[13] Stuckler D, Nestle M (2012). Big food, food systems, and global health. PLoS Med.

[14] Saloojee Y, Dagli E (2000). Tobacco industry tactics for resisting public policy on health. Bull World Health Organ.

[15] Brownell KD, Warner KE (2009). The perils of ignoring history: Big Tobacco played dirty and millions died. How similar is Big Food?. Milbank Q.

[16] Monteiro CA, Levy RB, Claro RM, de Castro IR, Cannon G (2011). Increasing consumption of ultra-processed foods and likely impact on human health: evidence from Brazil. Public Health Nutr.

[17] Moubarac J-C, Batal M, Martins APB (2013). Time trends changes in the consumption of ultra-processed products during the 20th century in Canada. Can J Diabetes.

[18] Silva V, Turci SRB, Oliveira APN, Richter AP (2017). Can the risk in public-private partnerships be classified?. Cad Saude Publica.

[19] Dube L, Labban A, Moubarac JC, Heslop G, Ma Y, Paquet C (2014). A nutrition/health mindset on commercial Big Data and drivers of food demand in modern and traditional systems. Ann N Y Acad Sci.

[20] Martinez S. Local Food Systems; Concepts, Impacts, and Issues. DIANE Publishing; 2010:87.

[21] Vedung E. Policy instruments: typologies and theories. In: Carrots, Sticks, and Sermons: Policy Instruments and Their Evaluation. UK: Transaction Publishers; 1998:21-58.

[22] Hood C, Margetts H. The Tools of Government in the Digital Age. Palgrave Macmillan; 2007:233.

[23] Jordan A, Wurzel RKW, Zito A (2005). The rise of ‘New’ Policy Instruments in comparative perspective: has governance eclipsed government?. Polit Stud.

[24] Gostin LO (2000). Public health law in a new century: part I: law as a tool to advance the community’s health. JAMA.

[25] Mello MM, Studdert DM, Brennan TA (2006). Obesity--the new frontier of public health law. N Engl J Med.

[26] WHO (2009). WHO Study Group on Tobacco Product Regulation: Report on the Scientific Basis of Tobacco Product Regulation: Third Report of a WHO Study Group.

[27] Hawkes C (2007). Regulating and litigating in the public interest: regulating food marketing to young people worldwide: trends and policy drivers. Am J Public Health.

[28] Magnusson R, Reeve B (2014). ‘Steering’ Private Regulation? A New Strategy for Reducing Population Salt Intake in Australia. Syd Law Rev.

[29] Mello MM, Pomeranz J, Moran P (2008). The interplay of public health law and industry self-regulation: the case of sugar-sweetened beverage sales in schools. Am J Public Health.

[30] Sharma LL, Teret SP, Brownell KD (2010). The food industry and self-regulation: standards to promote success and to avoid public health failures. Am J Public Health.

[31] Bexell M (2014). Global Governance, Legitimacy and (De)Legitimation. Globalizations.

[32] Carpenter D, Moss DA. Preventing Regulatory Capture: Special Interest Influence and How to Limit it. Cambridge: Cambridge University Press; 2013.

[33] World Health Organization. WHO’s engagement with non-State actors. Discussion paper for the informal consultation with Member States and non-State actors, 17-18 October 2013. Geneva: WHO; 2013.

[34] Chaib F (2012). WHO sets the record straight on work with the food and beverage industry. J Home Econ Inst Aust.

[35] Tschirley D, Reardon T, Dolislager M, Snyder J (2015). The Rise of a Middle Class in East and Southern Africa: Implications for Food System Transformation. J Int Dev.

[36] Ostrom E (2010). Beyond markets and states: polycentric governance of complex economic systems. Am Econ Rev.

[37] Hill PS (2011). Understanding global health governance as a complex adaptive system. Glob Public Health.

[38] Stoker G (1998). Governance as theory: five propositions. Int Soc Sci J.

[39] Hood C (2007). Intellectual Obsolescence and Intellectual Makeovers: Reflections on the Tools of Government after Two Decades. Governance.

[40] Jarvenpaa SL, Staples DS (2000). The use of collaborative electronic media for information sharing: an exploratory study of determinants. ‎J Strateg Inf Syst.

[41] Bodor JN, Rose D, Farley TA, Swalm C, Scott SK (2008). Neighbourhood fruit and vegetable availability and consumption: the role of small food stores in an urban environment. Public Health Nutr.

[42] Schermel A, Emrich TE, Arcand J, Wong CL, L’Abbe MR (2013). Nutrition marketing on processed food packages in Canada: 2010 Food Label Information Program. Appl Physiol Nutr Metab.

[43] Borys JM, Le Bodo Y, Jebb SA (2012). EPODE approach for childhood obesity prevention: methods, progress and international development. Obes Rev.

[44] Burt RS (1987). Social Contagion and Innovation: Cohesion versus Structural Equivalence. Am J Sociol.

[45] Borgatti SP, Obstfeld D, Davis J. Brokerage as a Process: Decoupling Third Party Action from Social Network Structure. In: Brass DJ, Labianca G, Mehra A, Halgin DS, Borgatti SP, eds. Contemporary Perspectives on Organizational Social Networks. Emerald Group Publishing Limited; 2014:135-159. Research in the Sociology of Organizations; vol. 40.

[46] Small ML. Unanticipated Gains: Origins of Network Inequality in Everyday Life. Oxford University Press; 2009:309.

[47] Stovel K, Shaw L (2012). Brokerage. Annu Rev Sociol.

[48] Cummins S, Flint E, Matthews SA (2014). New neighborhood grocery store increased awareness of food access but did not alter dietary habits or obesity. Health Aff (Millwood).

[49] Dubowitz T, Ghosh-Dastidar M, Cohen DA (2015). Diet and perceptions change with supermarket introduction in a food desert, but not because of supermarket use. Health Aff (Millwood).

[50] Abbott KW, Genschel P, Snidal D, Zangl B (2016). Two logics of indirect governance: Delegation and orchestration. Br J Polit Sci.

[51] Addy NA, Poirier A, Blouin C, Drager N, Dube L (2014). Whole-of-society approach for public health policymaking: a case study of polycentric governance from Quebec, Canada. Ann N Y Acad Sci.

